# Exploring Prostate Cancer Incidence Trends and Age Change in Cancer Registration Areas of Jiangsu Province, China, 2009 to 2019

**DOI:** 10.3390/curroncol31090408

**Published:** 2024-09-14

**Authors:** Hairong Zhou, Xin Hong, Weigang Miao, Weiwei Wang, Chenchen Wang, Renqiang Han, Jinyi Zhou

**Affiliations:** 1 Department of Non-Communicable Disease Prevention, Nanjing Municipal Center for Disease Control and Prevention, Nanjing 210009, China; zhouhrong@126.com (H.Z.); nj_hongxin@126.com (X.H.); nj_wangww@126.com (W.W.); isisccwang@163.com (C.W.); 2 Department of Non-Communicable Chronic Disease Control and Prevention Institute, Jiangsu Provincial Center for Disease Control and Prevention (Jiangsu Provincial Academy of Preventive Medicine), Nanjing 210009, China; 18705189723@163.com

**Keywords:** PCa, incidence, temporal trends, aging

## Abstract

(1) Background: Over the past few decades, Jiangsu Province, China, has witnessed a remarkable surge in the incidence of prostate cancer (PCa), accompanied by notable demographic shifts; (2) Methods: PCa data for Jiangsu Province from 2009 to 2019 were obtained from the Jiangsu Cancer Registry. We calculated crude and age-specific incidence rates (ASIRs), average age at onset, and age-specific composition ratios. Standardization was performed based on the Segi’s world population. Birth cohorts (1929–2019) were analyzed to assess PCa incidence by birth year. Trend analysis was conducted using the Joinpoint Regression Model, and average annual percent changes (AAPCs) with corresponding 95% confidence interval (CI) were computed. A linear regression model was used to analyze the relationship between the average age at diagnosis and calendar years; (3) Results: The ASIRs of PCa in Jiangsu Province increased significantly, with an AAPC of 11.25% (95%CI: 10.09%, 12.42%) from 2009 to 2019. This increase was observed across all age groups, particularly among those aged 0–59 years. Birth cohort analysis revealed a rising trend with earlier birth years showing higher incidence, notably in the 1959 cohort. In rural areas, the age-standardized average age at onset of PCa decreased from 75.45 years in 2009 to 73.39 years in 2019, and the peak age group shifted from 75–79 years in 2009 to 70–74 years in 2019; (4) Conclusions: The rising incidence of PCa in Jiangsu Province, along with an increased proportion of cases in younger age groups, highlights the need for targeted interventions.

## 1. Introduction

Prostate cancer (PCa) is currently the second most prevalent cancer among men worldwide and ranks fifth in cancer-related mortality among men [1]. According to the Global Cancer Statistics (GLOBOCAN 2022) by the International Agency for Research on Cancer, the estimated number of new PCa cases globally reached 1.47 million in 2022, accounting for 14.2% of all male malignancies. Although the incidence of PCa in China is relatively lower than the global average, significant increases have been observed over the past three decades [2,3,4,5]. The age-standardized incidence rate (ASIR) in China has risen substantially by approximately 2.3% per year from 1990 to 2019, reflecting a dramatic surge of 95.27% [6]. PCa has become the most common tumor affecting the male urogenital system in China, posing a significant threat to both the physical and mental health of the male population and emerging as a critical public health issue [7].

Established risk factors for PCa include aging, race, and family history [3]. Additionally, unhealthy lifestyle and dietary factors may also contribute to an increased risk of PCa [8,9]. The Human Development Index (HDI) has also been shown to impact the incidence and mortality of PCa [10,11].

Most epidemiological studies on PCa have focused on Europe and North America, with relatively few studies addressing the burden of PCa in different regions of China. Due to variations in economic development, medical advancements, lifestyle, and the extent of population aging across provinces, there are significant spatial differences in the burden of PCa [12]. Jiangsu Province, as a representative region of Eastern China and one of the most developed areas globally since the ‘Reform and Opening-up’ period, has experienced rapid economic growth and demographic changes. The proportion of individuals aged 65 and older in Jiangsu increased from 4.49% in 2010 to 15.38% in 2017, exceeding the United Nations’ threshold of 14% for a profoundly aging society. By 2020, the average life expectancy for males in Jiangsu had reached 77.02 years, an increase of 2.42 years since 2010, and surpassing the global average of 68 years. Given the acceleration of population aging and increasing life expectancy due to economic development, the outlook for PCa in Jiangsu requires further investigation.

Despite these demographic shifts, precise trends in PCa incidence rates and the average age at diagnosis in Jiangsu remain unclear. This study aims to describe recent trends in PCa incidence rates and age-specific characteristics from 2009 to 2019 in Jiangsu Province, providing a comprehensive view of age-related changes. By identifying key populations for PCa prevention and control, this research offers a theoretical foundation for understanding the current burden of PCa, guiding resource allocation, and enhancing the effectiveness of prevention and treatment strategies.

## 2. Materials and Methods

### 2.1. Study Design

This is a descriptive epidemiological study based on population-level cancer registry data from Jiangsu Province, designed to analyze trends and age-related changes in prostate cancer incidence between 2009 and 2019. Data were obtained from the Jiangsu Provincial Center for Disease Control and Prevention (CDC), which oversees the collection, quality evaluation, and publication of cancer registration data from local population-based cancer registries. Cancer diagnoses are reported to local registries from multiple sources, including hospitals, community health centers, the Urban Resident Basic Medical Insurance program, and the New Rural Cooperative Medical Scheme. Data items included in the analysis were demographics (date of birth or age, area of residents), and tumor characteristics (date of diagnosis, histology). The analysis used the most recent data from 16 population-based cancer registries, covering approximately 17.3 million people, or about 22.15% of Jiangsu’s total population.

The data analysis followed a three-step process: first, we calculated crude and age-specific incidence rates, the average age at onset, and age-specific composition ratios. Standardization was performed using Segi’s world population. Second, we constructed birth cohorts (1929–2019) to assess prostate cancer incidence by birth year. Third, we examined temporal trends in incidence rates from 2009 to 2019 by fitting joinpoint models and used a linear regression model to explore the relationship between the average age at onset and calendar year. All analyses were stratified by geographic areas and age groups.

### 2.2. Data Source and Quality Control

Cancer incidence data from 2009 to 2019 were obtained from the Jiangsu Provincial Center for Disease Control and Prevention, which oversees the collection, quality evaluation, and publication of cancer registration data from local population-based cancer registries in Jiangsu Province, China. Data quality was assessed according to the guidelines for Chinese Cancer Registration and criteria set by the International Agency for Research on Cancer/International Association of Cancer Registries (IARC/IACR) [13,14]. Quality control indicators included the mortality-to-incidence ratio (M/I), the proportion of morphological verification (MV%), the percentage of cases identified with death certification only (DCO%), and the stability of cancer incidence. The study included data from 16 cancer registries covering the years 2009 to 2019, with a total population of 17,394,974 (8,813,575 males and 8,581,399 females), representing approximately 22.15% of Jiangsu’s total population (78,515,666) in 2019. These registries comprised 7 urban and 9 rural areas, with populations of 9,702,072 and 7,692,902, respectively. Data from 7 registries were accepted for publication in *Cancer Incidence in Five Continents (CI5) Volume XII*. All registration data were coded according to the *International Classification of Diseases for Oncology, 3rd edition* (ICD-O-3) [15] and the *International Statistical Classification of Diseases 10th Revision* (ICD-10) [16], with prostate cancer coded as C61 (ICD-10). Data used in this study do not contain personal information, thus ethical approval was not required. The participating registry has given its consent. The study was performed in accordance with the Declaration of Helsinki.

### 2.3. Statistical Analysis

#### 2.3.1. Incidence Trend Analysis

PCa incidence rates from 2009 to 2019 were calculated by sex, age group (0, 1–4, 5–80 in 5-year increments, and 85+), and region (urban and rural). Age-standardized rates were calculated using Segi’s world population. Given the relatively low incidence under age 60, patients were grouped into four age categories (0–59, 60–69, 70–79, and ≥80 years) to compare incidence rate changes. The average annual percentage change (AAPC) and its 95% confidence interval (CI) were determined using Joinpoint Regression Software (Version 4.7.0.0) [17]. To prevent erroneous trend change results, when analyzing with Joinpoint software, all models were set to a maximum of 2 joinpoints (i.e., the trend was divided into at most 3 segments). Trends in crude rates, age-standardized rates, and age-specific rates were classified as increasing or decreasing if the AAPC was statistically significant (two-sided *p* < 0.05); otherwise, trends were considered stable. Birth cohort analysis was conducted for patients born between 1929 and 2019, evaluating age-specific incidence rates and trends by birth year.

#### 2.3.2. Analysis of Age Characteristics

The age of onset and age-specific incidence composition of PCa were calculated using frequency tables [18]. Standardization was performed using Segi’s world population. A linear regression model was used to examine the relationship between the average age of onset and calendar year. The formula used was *y* = a + β*x* + e, where *y* is the average age or composition, a is the constant term, β is the regression coefficient, and e is the random error term. The regression coefficient β represents the average annual change. Statistical significance of β was assessed using T-tests, with *p* < 0.05 indicating statistical significance. The trend in the composition ratio of PCa in males aged 60 and older was analyzed using Joinpoint Regression Software (Version 4.7.0.0).

## 3. Results

### 3.1. Trend in PCa Incidence

Population size and structure by area, year, and age in Jiangsu Province between 2009 and 2019 are shown in Table 1 and Table 2. Table 3 presents the temporal trends in PCa incidence in Jiangsu Province from 2009 to 2019. The crude incidence rate of PCa exhibited a substantial increase, rising from 4.60 per 100,000 in 2009 to 18.47 per 100,000 in 2019, with an average annual percentage change (AAPC) of 14.56% (95% CI: 12.83%, 16.31%). Urban areas saw an increase from 6.25 per 100,000 in 2009 to 20.56 per 100,000 in 2019, with an AAPC of 12.61% (95% CI: 10.16%, 15.10%). Rural areas demonstrated a greater rise, from 2.81 per 100,000 in 2009 to 15.88 per 100,000 in 2019, with an AAPC of 18.10% (95% CI: 16.90%, 19.31%), indicating a faster growth rate in rural areas compared to urban areas.

After age standardization, the increase in age-standardized incidence rates (ASIRs) was moderated, with an AAPC of 11.25% (95% CI: 10.09%, 12.42%). The ASIRs rose significantly in both urban (AAPC = 9.51%, 95% CI: 6.71%, 12.38%) and rural regions (AAPC = 14.83%, 95% CI: 13.49%, 16.19%), with rural areas continuing to experience a faster growth rate than urban areas.

### 3.2. Age-Specific Incidence and Birth Cohort Analysis

**Figure 1:**
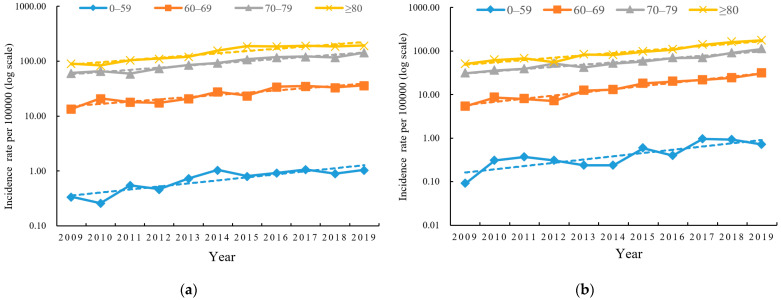
Incidence of prostate cancer by age groups in different regions in Jiangsu Province, 2009–2019: (**a**) urban areas; (**b**) rural areas.

In Jiangsu Province, PCa incidence was relatively low before age 60, with a marked increase thereafter, peaking at age 80 and older (Table 4, Figure 1). This pattern was observed in both urban and rural areas. From 2009 to 2019, all age groups experienced an upward trend, with rural areas showing a more pronounced increase compared to urban areas. The 0–59 age group saw the most significant rise in incidence in both settings, while the 70–79 age group had the slowest increase in rural areas, and the ≥80 age group had the slowest increase in urban areas.

**Figure 2:**
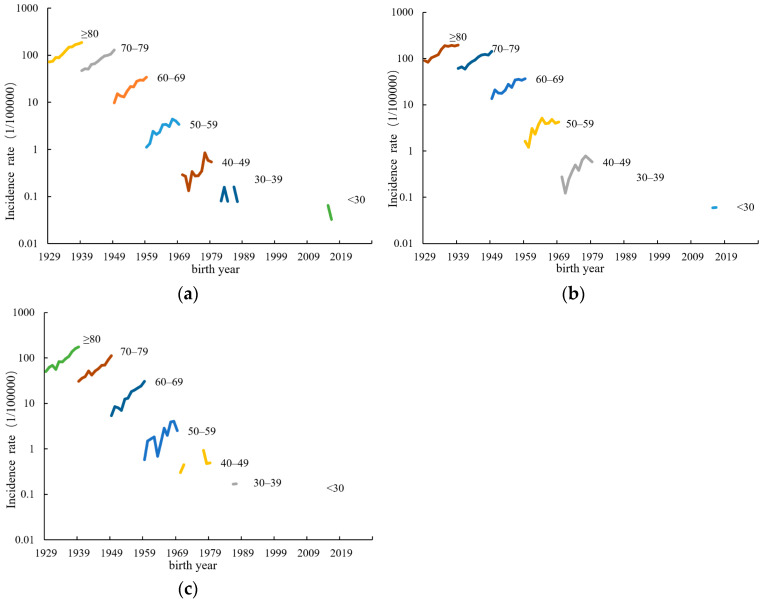
Incidence of prostate cancer in the birth cohort of different regions in Jiangsu Province, 2009–2019: (**a**) total; (**b**) urban areas; (**c**) rural areas.

Birth cohort analysis revealed that individuals born in later years within the same age groups faced higher risks of PCa compared to those born earlier, with the greatest impact observed in the 50–59 age group. Rural areas exhibited a more rapid increase in PCa incidence rates among individuals aged 50 and above compared to urban areas (Figure 2).

### 3.3. Analysis of Age-Related Changes in PCa Incidence

Table 5 illustrates that the average age at onset of PCa in Jiangsu Province remained relatively stable from 2009 to 2019. Age-standardized data reveal a decrease in the average age at onset in rural areas, from 75.45 years in 2009 to 73.39 years in 2019, with an annual change of −0.149 years. No significant correlation was found between standardized onset age and the years overall or in urban areas.

**Figure 3:**
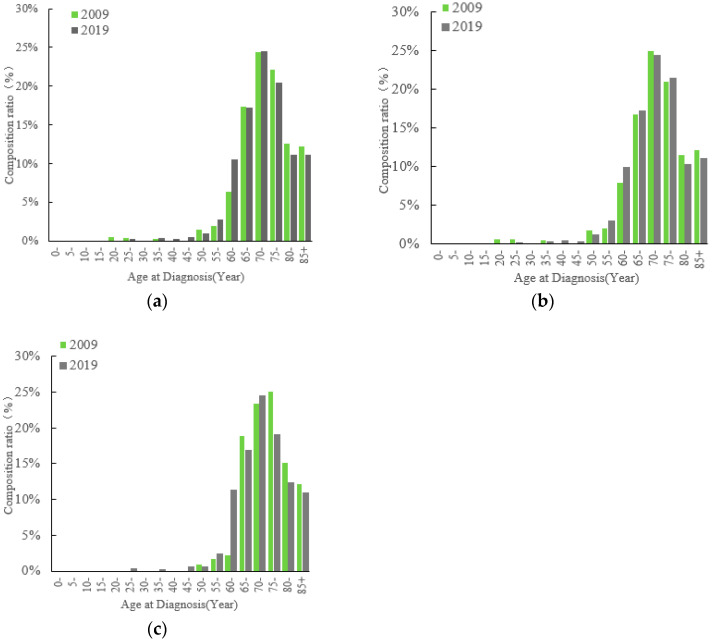
Standardized age-specific composition ratio of prostate cancer incidence Jiangsu Province in 2009 and 2019: (**a**) total; (**b**) urban areas; (**c**) rural areas. Age-standardized composition ratio was standardized by Segi’s World population.

Comparing 2019 to 2009, the proportion of PCa cases standardized by age increased for the 55–64 age group but decreased for those over 75 (Figure 3). In urban areas, the standardized incidence proportions for ages 50–54, 70–74, and ≥80 decreased, while they increased for the 55–69 and 75–79 age groups. Conversely, in rural areas, the peak age group shifted from 75–79 years to 70–74 years, with a significant increase in the 55–64 age group and a decrease in those aged 75 and above.

From 2009 to 2019, PCa incidence in Jiangsu Province was predominantly in individuals aged 60 and above, accounting for over 90% of all cases (Table 6). The proportion of cases in this age group remained stable before and after age standardization, with no statistically significant differences observed.

## 4. Discussion

This study explores the trends in prostate cancer (PCa) incidence and age-specific changes in Jiangsu from 2009 to 2019, uncovering several significant insights.

The increase in crude incidence rates and age-standardized incidence rates (ASIRs) suggests that changes in population age structure and growth had minimal impact on this rise. In contrast to decreasing incidence trends observed in the United States, Canada, and some European countries [19,20,21], Jiangsu has seen a more rapid increase in ASIR, consistent with findings in other Asian regions such as Guangzhou [22], Japan [23], and Korea [24]. Jiangsu, a leading economic region in China with a GDP of 121,300 RMB in 2020, has experienced significant social and economic improvements over the past four decades. Changes in dietary patterns and lifestyle due to these advancements likely contribute to the observed increase in PCa incidence. Medical imaging and other examination methods will also affect the incidence.

Genetic mutations are considered a primary driver of PCa, which may explain the lower incidence rates among Asian men [25]. However, significant geographical variations within Asia exist. Despite similarities in genetic backgrounds and cultures, Jiangsu has lower PCa incidence rates compared to Japan and South Korea [26,27]. The Westernization of diets and unhealthy lifestyles may partially explain Japan’s doubled incidence of latent PCa from 1983 to 2013 [28]. Additionally, earlier PSA-based screening, improved medical access, and increased public awareness could contribute to these differences [29,30].

From 2009 to 2019, a higher incidence of PCa was observed in urban compared to rural areas, potentially due to higher obesity rates, unhealthy behaviors, and economic disparities in urban settings. However, the gap between urban and rural areas has been narrowing, aligning with trends observed in other cities [31,32]. This convergence is attributed to three main factors: the spread of economic development to rural areas, leading to urbanized lifestyles; advancements in medical technology and PCa screening in rural areas; and improved quality of cancer registration data in these areas.

A noticeable rise in PCa incidence was found across all age groups, with the 0–59 age group showing the fastest increase in both urban and rural areas. The standardized incidence composition for the 60–64 age group increased, while the incidence in those aged 75 and older decreased. In rural areas, the average age at diagnosis decreased from 75.45 years in 2009 to 73.39 years in 2019. Similar trends have been observed in developed countries, where younger age groups are experiencing faster increases in incidence rates [4,33,34,35].

Traditionally considered a disease of older age, PCa is now showing an “early-onset cancer epidemic,” with rising cases among younger men [36]. Data from SEER and IHME GBD indicate increasing incidence rates in men aged 15–39 in various regions [35]. Globally, the incidence of early-onset PCa in men aged 15 to 49 has shown the fastest growth, with an estimated annual percentage change (EAPC) of 2.23% from 1990 to 2019 [34], similar to our findings. The widespread use of PSA screening has led to earlier detection of tumors [37,38,39,40], although early-onset PCa, which is often more aggressive and with poorer prognosis, may not be fully explained by PSA testing alone [41].

Changes in diet, lifestyle, and environment since the 20th century might impact cancer risk [42]. A study found that about 45.2% of cancer deaths in China are attributable to modifiable risk factors [43]. Despite a slight decline in smoking rates, passive smoking has increased, and obesity and diabetes rates have risen significantly (50.7% to 47.7% for smoking, 12.6% to 17.2% for obesity, 6.7% to 15.0% for diabetes). Increasing intake of animal products, obesity, and unhealthy habits contribute to PCa risk. The rising incidence in successive birth cohorts, especially among those aged 50 and older, underscores the need for further research and increased attention to PCa among younger men [44].

Although the incidence of PCa is rising among those under 60, over 90% of cases are still in individuals aged 60 and older, consistent with findings in high Human Development Index (HDI) countries [45]. The cumulative effects of carcinogenic factors, aging, genetic mutations, and immune system decline significantly increase PCa risk [46]. As Jiangsu continues to age and life expectancy increases, the future burden of PCa will likely rise. Therefore, more focus should be placed on PCa prevention and control among the elderly.

This study benefits from data derived from the population-based Jiangsu Cancer Registry, which enhances its generalizability. The progress and development in cancer registration in Jiangsu over recent years have contributed to the robustness of our findings. However, there are notable limitations. First, we lacked information on cancer stage, grade and life-state follow-up-related information, which are essential for a more comprehensive interpretation of our results. Second, the study depended on cancer registry data, which did not include individual risk factor information or population-level screening data. These limitations should be considered when interpreting the findings of our study.

## 5. Conclusions

The burden of prostate cancer in Jiangsu appears to be shifting towards that of high-income countries, with notable age-related changes influenced by factors such as population aging, risk factor exposures, and evolving screening practices. Jiangsu has introduced several health initiatives aimed at enhancing cancer control and prevention, including the Medium-to-Long-Term Plan for Prevention and Control of Chronic Diseases in Jiangsu (2017–2025) and the Healthy Jiangsu 2030 program. The implementation of these plans is expected to impact age-specific incidence rates, particularly among males.

It is crucial to increase awareness among both the public and healthcare professionals about the trend towards earlier onset of prostate cancer in rural areas and the high prevalence among older individuals. Future efforts should focus on comprehensive health education, adherence to screening recommendations, lifestyle modifications, and continued research into the mechanisms of prostate cancer. These measures are essential for improving the health outcomes in Jiangsu Province.

## Figures and Tables

**Table 1:** Population structure by area and year in Jiangsu Province during 2009–2019.

Year	Total	Urban Areas	Rural Areas
2009	8,019,671	4,174,296	3,845,375
2010	8,096,710	4,216,054	3,880,656
2011	8,522,229	4,642,339	3,879,890
2012	8,563,669	4,664,893	3,898,776
2013	8,700,871	4,713,718	3,987,153
2014	8,760,946	4,759,389	4,001,557
2015	8,787,737	4,775,387	4,012,350
2016	8,830,525	4,807,068	4,023,457
2017	8,832,074	4,820,577	4,011,497
2018	8,800,171	4,847,542	3,952,629
2019	8,813,575	4,883,057	3,930,518

**Table 2:** Population structure by age in Jiangsu Province during 2009–2019.

Age	Total	Urban Areas	Rural Areas
0–	876,834	507,130	369,704
1–4	3,888,093	2,251,306	1,636,787
5–9	5,190,691	2,851,884	2,338,807
10–14	5,197,631	2,658,435	2,539,196
15–19	5,126,747	2,628,907	2,497,840
20–24	6,015,093	3,224,387	2,790,706
25–29	7,212,431	3,898,368	3,314,063
30–34	7,092,290	3,836,622	3,255,668
35–39	6,703,990	3,516,748	3,187,242
40–44	7,326,266	3,928,026	3,398,240
45–49	8,308,845	4,516,319	3,792,526
50–54	7,597,445	4,031,210	3,566,235
55–59	6,289,065	3,384,198	2,904,867
60–64	5,824,887	3,305,588	2,519,299
65–69	4,387,011	2,483,050	1,903,961
70–74	3,181,717	1,745,891	1,435,826
75–79	2,255,873	1,250,760	1,005,113
80–84	1,391,122	790,115	601,007
85+	862,147	495,376	366,771

**Table 3:** Trend of prostate cancer incidence rate in different regions in Jiangsu Province, 2009–2019.

Year	Crude Rate (1/105)			ASIR (1/105)		
	Total	Urban Areas	Rural Areas	Total	Urban Areas	Rural Areas
2009	4.60	6.25	2.81	2.88	3.83	1.78
2010	5.76	7.40	3.97	3.39	4.27	2.39
2011	6.23	7.84	4.30	3.54	4.31	2.51
2012	7.02	8.98	4.67	3.82	4.69	2.65
2013	8.37	10.67	5.64	4.32	5.42	2.95
2014	10.14	13.34	6.32	5.19	6.65	3.33
2015	11.74	14.87	8.03	5.78	7.05	4.17
2016	13.57	17.16	9.27	6.62	8.17	4.65
2017	14.96	18.21	11.04	7.15	8.51	5.40
2018	15.83	17.84	13.36	7.34	8.09	6.36
2019	18.47	20.56	15.88	8.38	9.13	7.41
AAPC (%)	14.56	12.61	18.10	11.25	9.51	14.83
95% CI	12.83, 16.31	10.16, 15.10	16.90, 19.31	10.09, 12.42	6.71, 120.38	13.49, 160.19
*p*	<0.05	<0.05	<0.05	<0.05	<0.05	0.05

Abbreviations: ASIR, age standardized incidence rate by World Segi’ s population; AAPC, Average annual percentage change.

**Table 4:** Trend of age-specific incidence rate of prostate cancer in Jiangsu Province, 2009–2019.

Year	0–59 years			60–69 years			70–79 years			≥80 years		
	Total	Urban Areas	Rural Areas	Total	Urban Areas	Rural Areas	Total	Urban Areas	Rural Areas	Total	Urban Areas	Rural Areas
2009	0.22	0.34	0.09	9.72	13.47	5.38	47.18	61.38	30.75	72.04	89.85	50.26
2010	0.28	0.26	0.31	15.20	21.03	8.47	51.48	65.44	35.78	74.14	83.69	62.35
2011	0.47	0.55	0.37	13.59	18.00	7.91	50.55	59.54	39.10	89.35	103.76	68.23
2012	0.40	0.47	0.31	13.03	17.54	7.05	63.79	73.56	51.23	88.98	111.68	55.78
2013	0.51	0.73	0.24	17.08	20.68	12.34	65.44	84.93	42.04	104.46	120.46	83.60
2014	0.68	1.05	0.24	21.47	27.72	13.05	74.15	92.85	51.47	123.90	155.82	81.94
2015	0.71	0.81	0.58	21.29	23.65	18.12	85.65	108.56	57.95	148.48	188.28	95.85
2016	0.68	0.92	0.40	28.06	34.21	19.75	96.52	119.52	68.84	151.34	184.75	107.72
2017	1.01	1.06	0.96	29.81	35.65	21.79	99.22	122.78	70.23	167.88	191.15	138.28
2018	0.91	0.90	0.92	29.64	33.62	24.32	106.50	117.97	91.71	176.40	187.33	161.78
2019	0.90	1.05	0.71	33.91	36.31	30.72	130.01	144.14	111.73	186.29	194.16	175.48
AAPC (%)	15.07	13.59	18.78	12.58	9.87	18.22	10.61	9.53	12.43	11.04	8.46	13.20
95% CI	10.80, 19.52	7.55, 19.98	8.66, 29.84	9.76, 15.46	6.71, 13.13	14.68, 21.88	9.42, 11.82	7.67, 11.42	10.05, 14.86	9.56, 12.54	1.28, 16.14	10.49, 15.97
*p*	<0.05	<0.05	<0.05	<0.05	<0.05	<0.05	<0.05	<0.05	<0.05	<0.05	<0.05	<0.05

Abbreviation: AAPC, Average annual percentage change.

**Table 5:** Average age at diagnosis of prostate cancer in Jiangsu Province, 2009–2019.

Year	Average Age at Diagnosis (Years)			Standardized Average Age at Diagnosis (Years)		
	Total	Urban Areas	Rural Areas	Total	Urban Areas	Rural Areas
2009	75.02	74.59	76.06	74.05	73.43	75.45
2010	74.27	74.04	74.74	73.13	72.76	73.86
2011	74.58	74.60	74.54	73.51	73.37	73.63
2012	75.07	75.05	75.11	73.98	73.74	74.32
2013	74.92	74.78	75.26	73.45	73.40	73.56
2014	74.66	74.37	75.41	73.17	72.77	74.12
2015	75.20	75.65	74.21	73.65	74.12	72.56
2016	74.77	74.74	74.86	73.15	73.05	73.31
2017	74.63	74.58	74.72	73.02	73.11	72.93
2018	74.83	74.77	74.93	73.46	73.36	73.62
2019	74.75	74.72	74.78	73.32	73.25	73.39
β	0.006	0.034	−0.060	−0.048	0.001	−0.149
t	0.247	0.887	−1.331	−1.604	0.019	−2.547
*p*	0.811	0.398	0.216	0.143	0.985	0.031

Abbreviations: β, Annual growth of incidence composition, statistical analysis by *t*-test; Standardized average age at diagnosis, age-standardized rate standardized by Segi’s World standard population.

**Table 6:** Composition ratio of prostate cancer in males above 60 years old in Jiangsu Province, 2009–2019.

Year	Composition Ratio			Age-Standardized Composition Ratio		
	Total	Urban Areas	Rural Areas	Total	Urban Areas	Rural Areas
2009	95.93	95.40	97.22	95.28	94.40	97.17
2010	95.92	97.12	93.51	95.58	96.81	93.07
2011	93.79	94.23	92.81	93.15	93.52	92.11
2012	95.34	95.70	94.51	94.81	95.05	94.11
2013	95.05	94.43	96.44	94.07	93.45	95.46
2014	94.59	93.70	96.84	93.51	92.18	96.39
2015	95.16	95.63	94.10	93.78	94.15	92.85
2016	95.99	95.76	96.51	94.66	94.05	95.68
2017	94.63	95.44	93.00	92.87	93.81	91.03
2018	95.48	96.07	94.51	94.02	94.44	93.26
2019	96.19	96.02	96.47	94.84	94.39	95.42
AAPC%	0.03	0.04	0.03	−0.10	−0.09	−0.10
95%CI	−0.14, 0.21	−0.19, 0.27	−0.37, 0.42	−0.29, 0.10	−0.36, 0.17	−0.56, 0.36
*p*	0.68	0.72	0.89	0.30	0.44	0.63

Abbreviations: Age-standardized composition ratio: standardized by Segi’s World standard population. AAPC, Average annual percentage change

## Data Availability

The data that support the findings of this study are available from the corresponding author upon reasonable request.

## References

[1] Bray F., Laversanne M., Sung H., Ferlay J., Siegel R.L., Soerjomataram I., Jemal A. (2024). Global cancer statistics 2022: GLOBOCAN estimates of incidence and mortality worldwide for 36 cancers in 185 countries. CA Cancer J. Clin..

[2] He H., Liang L., Han D., Xu F., Lyu J. (2022). Different Trends in the Incidence and Mortality Rates of Prostate Cancer between China and the USA: A Joinpoint and Age-Period-Cohort Analysis. Front. Med..

[3] Wang L., Lu B., He M., Wang Y., Wang Z., Du L. (2022). Prostate Cancer Incidence and Mortality: Global Status and Temporal Trends in 89 Countries From 2000 to 2019. Front. Public Health.

[4] Zhou C.K., Check D.P., Lortet-Tieulent J., Laversanne M., Jemal A., Ferlay J., Bray F., Cook M.B., Devesa S.S. (2015). Prostate cancer incidence in 43 populations worldwide: An analysis of time trends overall and by age group. Int. J. Cancer.

[5] Han B., Zheng R., Zeng H., Wang S., Sun K., Chen R., Li L., Wei W., He J. (2024). Cancer incidence and mortality in China, 2022. J. Natl. Cancer Cent..

[6] Wang F., Wang C., Xia H., Lin Y., Zhang D., Yin P., Yao S. (2022). Burden of Prostate Cancer in China, 1990–2019: Findings from the 2019 Global Burden of Disease Study. Front. Endocrinol..

[7] Tian Y.Q., Yang J.C., Hu J.J., Ding R., Ye D.W., Shang J.W. (2023). Trends and risk factors of global incidence, mortality, and disability of genitourinary cancers from 1990 to 2019: Systematic analysis for the Global Burden of Disease Study 2019. Front. Public Health.

[8] Støyten M., Knutsen T., Stikbakke E., Agledahl I., Wilsgaard T., Eggen A.E., Richardsen E., Giovannucci E., Thune I., Haugnes H.S. (2024). Excess weight, weight gain, and prostate cancer risk and prognosis: The PROCA-life study. Acta Oncol..

[9] Zhang H., Huang D., Zhang Y., Wang X., Wu J., Hong D. (2023). Global burden of prostate cancer attributable to smoking among males in 204 countries and territories, 1990–2019. BMC Cancer.

[10] Fathollahi F., Khazaei Z., Abbasi M., Goodarzi E. (2023). Burden of prostate cancer and relationship with the human development index (HDI) in Asia: A study of Global Burden disease in 2019. Casp. J. Intern. Med..

[11] Bray F., Jemal A., Grey N., Ferlay J., Forman D. (2012). Global cancer transitions according to the Human Development Index (2008–2030): A population-based study. Lancet Oncol..

[12] Liu X., Zhou M., Wang F., Mubarik S., Wang Y., Meng R., Shi F., Wen H., Yu C. (2020). Secular Trend of Cancer Death and Incidence in 29 Cancer Groups in China, 1990–2017: A Joinpoint and Age-Period-Cohort Analysis. Cancer Manag. Res..

[13] Bray F., Parkin D.M. (2009). Evaluation of data quality in the cancer registry: Principles and methods. Part I: Comparability, validity and timeliness. Eur. J. Cancer.

[14] Parkin D.M., Bray F. (2009). Evaluation of data quality in the cancer registry: Principles and methods Part II. Completeness. Eur. J. Cancer.

[15] Jack A., Percy C., Sobin L., Whelan S. (2000). International Classification of Diseases for Oncology.

[16] World Health Organization (2016). International Statistical Classification of Diseases and Related Health Problems 10th Version. https://icd.who.int/browse10/2019/en.

[17] Kim H.J., Fay M.P., Feuer E.J., Midthune D.N. (2000). Permutation tests for joinpoint regression with applications to cancer rates. Stat. Med..

[18] Bidoli E., Lamaj E., Angelin T., Forgiarini O., De Santis E., Serraino D. (2021). Linearity of Age at Cancer Onset Worldwide: 25-Year Population-Based Cancer Registry Study. Cancers.

[19] Siegel R.L., Miller K.D., Wagle N.S., Jemal A. (2023). Cancer statistics, 2023. CA Cancer J. Clin..

[20] Everatt R., Gudavičienė D. (2022). An analysis of time trends in breast and prostate cancer mortality rates in Lithuania, 1986–2020. BMC Public Health.

[21] Arık A., Dodd E., Streftaris G. (2020). Cancer morbidity trends and regional differences in England—A Bayesian analysis. PLoS ONE.

[22] Luo A., Dong H., Lin X., Liao Y., Liang B., Chen L., Lin G., Hao Y. (2021). Time trends of major cancers incidence and mortality in Guangzhou, China 2004–2015: A Joinpoint and Age-Period-Cohort Analysis. Cancer Med..

[23] Sun D., Cao M., Li H., He S., Chen W. (2020). Cancer burden and trends in China: A review and comparison with Japan and South Korea. Chin. J. Cancer Res..

[24] Lee H.Y., Kim D.K., Doo S.W., Yang W.J., Song Y.S., Lee B., Kim J.H. (2020). Time Trends for Prostate Cancer Incidence from 2003 to 2013 in South Korea: An Age-Period-Cohort Analysis. Cancer Res. Treat..

[25] Cook L.S., Goldoft M., Schwartz S.M., Weiss N.S. (1999). Incidence of adenocarcinoma of the prostate in Asian immigrants to the United States and their descendants. J. Urol..

[26] Zhu Y., Mo M., Wei Y., Wu J., Pan J., Freedland S.J., Zheng Y., Ye D. (2021). Epidemiology and genomics of prostate cancer in Asian men. Nat. Rev. Urol..

[27] Ju W., Zheng R., Zhang S., Zeng H., Sun K., Wang S., Chen R., Li L., Wei W., He J. (2023). Cancer statistics in Chinese older people, 2022: Current burden, time trends, and comparisons with the US, Japan, and the Republic of Korea. Sci. China Life Sci..

[28] Kimura T., Takahashi H., Okayasu M., Kido M., Inaba H., Kuruma H., Yamamoto T., Furusato B., Furusato M., Wada T. (2016). Time trends in histological features of latent prostate cancer in Japan. J. Urol..

[29] Kye S.Y., Lee M.H., Yoo J., Oh K.H., Jun J.K. (2017). Factors affecting satisfaction with cancer information provided through the social networking services of the National Cancer Information Center in Korea. Epidemiol. Health.

[30] Okuhara T., Ishikawa H., Urakubo A., Hayakawa M., Yamaki C., Takayama T., Kiuchi T. (2018). Cancer information needs according to cancer type: A content analysis of data from Japan’s largest cancer information website. Prev. Med. Rep..

[31] Li X., Deng Y., Tang W., Sun Q., Chen Y., Yang C., Yan B., Wang Y., Wang J., Wang S. (2018). Urban-Rural Disparity in Cancer Incidence, Mortality, and Survivals in Shanghai, China, during 2002 and 2015. Front. Oncol..

[32] Yuan S., Xie S.H. (2021). Urban-rural disparity in cancer incidence in China, 2008–2012: A cross-sectional analysis of data from 36 cancer registers. BMJ Open.

[33] Newby J.A., Busby C.C., Howard C.V., Platt M.J. (2007). The cancer incidence temporality index: An index to show temporal changes in the age of onset of overall and specific cancer (England and Wales, 1971–1999). Biomed. Pharmacother..

[34] Zhao J., Xu L., Sun J., Song M., Wang L., Yuan S., Zhu Y.S., Wan Z.W., Larsson S., Tsilidis K. (2023). Global trends in incidence, death, burden and risk factors of early-onset cancer from 1990 to 2019. BMJ Oncol..

[35] Bleyer A., Spreafico F., Barr R. (2020). Prostate cancer in young men: An emerging young adult and older adolescent challenge. Cancer.

[36] Salinas C.A., Tsodikov A., Ishak-Howard M., Cooney K.A. (2014). Prostate cancer in young men: An important clinical entity. Nat. Rev. Urol..

[37] Martin R.M., Turner E.L., Young G.J., Metcalfe C., Walsh E.I., Lane J.A., Sterne J.A.C., Noble S., Holding P., Ben-Shlomo Y. (2024). Prostate-Specific Antigen Screening and 15-Year Prostate Cancer Mortality: A Secondary Analysis of the CAP Randomized Clinical Trial. JAMA.

[38] Fenton J.J., Weyrich M.S., Durbin S., Liu Y., Bang H., Melnikow J. (2018). Prostate-Specific Antigen-Based Screening for Prostate Cancer: Evidence Report and Systematic Review for the US Preventive Services Task Force. JAMA.

[39] Draisma G., De Koning H.J. (2003). MISCAN: Estimating lead-time and over-detection by simulation. BJU Int..

[40] Shao Y.H., Demissie K., Shih W., Mehta A.R., Stein M.N., Roberts C.B., Dipaola R.S., Lu-Yao G.L. (2009). Contemporary risk profile of prostate cancer in the United States. JNCI J. Natl. Cancer Inst..

[41] Lange E.M., Salinas C.A., Zuhlke K.A., Ray A.M., Wang Y., Lu Y., Ho L.A., Luo J., Cooney K.A. (2012). Early onset prostate cancer has a significant genetic component. Prostate.

[42] Zi H., He S.H., Leng X.Y., Xu X.F., Huang Q., Weng H., Zhu C., Li L.Y., Gu J.M., Li X.H. (2021). Global, regional, and national burden of kidney, bladder, and prostate cancers and their attributable risk factors, 1990–2019. Mil. Med. Res..

[43] Chen W., Xia C., Zheng R., Zhou M., Lin C., Zeng H., Zhang S., Wang L., Yang Z., Sun K. (2019). Disparities by province, age, and sex in site-specific cancer burden attributable to 23 potentially modifiable risk factors in China: A comparative risk assessment. Lancet Glob. Health.

[44] Li J., Djenaba J.A., Soman A., Rim S.H., Master V.A. (2012). Recent trends in prostate cancer incidence by age, cancer stage, and grade, the United States, 2001–2007. Prostate Cancer.

[45] Zhang W., Cao G., Wu F., Wang Y., Liu Z., Hu H., Xu K. (2023). Global Burden of Prostate Cancer and Association with Socioeconomic Status, 1990–2019: A Systematic Analysis from the Global Burden of Disease Study. J. Epidemiol. Glob. Health.

[46] Pilleron S., Soto-Perez-de-Celis E., Vignat J., Ferlay J., Soerjomataram I., Bray F., Sarfati D. (2021). Estimated global cancer incidence in the oldest adults in 2018 and projections to 2050. Int. J. Cancer.

